# 

**Published:** 1948

**Authors:** 


### CONTENTS

| | Page |
|---|---|
| **Cerebral Diplegia following Birth Injury.** By R. M. NORMAN, M.D. | 43 |
| **Thyroidectomy or Thiouracil?** By A. RENDLE SHORT, M.D., B.S., B.Sc., F.R.C.S. .. .. | 48 |
| **Factors in the Ætiology of Disseminated Sclerosis.** By A. M. G. CAMPBELL, D.M., M.R.C.P. .. .. .. | 54 |
| **Reviews of Books** .. .. .. .. .. .. .. .. | 58 |
| **Editorial Notes** .. .. .. .. .. .. .. .. | 61 |
| **Obituary:** DAVID ALFRED ALEXANDER, M.B., CH.B. (Victoria) .. | 62 |
| **Meetings of Societies** .. .. .. .. .. .. .. | 63 |
| **The Medical Library of the University of Bristol** .. .. .. .. | 64 |
| **Publications Received** .. .. .. .. .. .. .. | 66 |
| **Local Medical Notes** .. .. .. .. .. .. .. | 66 |

